# Application of 3D-Woven Fabrics for Packaging Materials for Terminally Sterilized Medical Devices

**DOI:** 10.3390/polym14224952

**Published:** 2022-11-16

**Authors:** Stana Kovačević, Beti Rogina-Car, Ana Kiš

**Affiliations:** 1 Department of Textile Design and Management, Faculty of Textile Technology, University of Zagreb, Prilaz baruna Filipovi’ca 28a, 10000 Zagreb, Croatia; 2 Department of Clothing Technology, Faculty of Textile Technology, University of Zagreb, Prilaz brauna Filipovića 28a, 10000 Zagreb, Croatia; 3 Vertiv Croatia d.o.o., Oreškovićeva ulica 6n/2, 10000 Zagreb, Croatia

**Keywords:** 3D woven fabric, packaging material, sterilization, microbial barrier, ball bursting test

## Abstract

This research aimed to test a newly developed 3D fabric for use in a hospital sterilization unit as a packaging material. Two basic properties were tested: the efficiency of the microbial barrier, and the bursting strength of the woven fabric, determined with a steel ball. Material deformations caused by bursting are common in medical sterilization, as a consequence of the packaging of the medical tools needed in surgery. Six 3D-fabric samples were woven from the same warp, with three weft densities and in two different weaves. The weaving conditions and other construction characteristics of the fabrics were the same. To determine the effectiveness of the microbial barrier, bacterial endospores of an apathogenic species of the genus *Bacillus, Geobacillus stearothermophilus* and *Bacillus atrophaeus*, were used. Mechanical testing of the 3D-woven fabric, i.e., the bursting strength of the fabric using a steel ball, was carried out according to the standard method. The results showed the exceptional puncture strength of the woven fabrics and their formation of an effective microbial barrier, i.e., complete impermeability to microorganisms in five samples, which is the main condition for possible use as a packaging material in medical sterilization. Sample 3tp did not provide an effective microbial barrier and did not meet the basic requirements for use in medical sterilization.

## 1. Introduction

There have been significant developments in the textile industry in recent times based on the production of technical fabrics. The range of technical fabrics is very wide. It includes a variety of products and, therefore, it is difficult to find a comprehensive definition of technical fabrics. Whether a woven fabric is a technical fabric primarily depends on its use, sometimes on the production technology, or on the raw material composition [1]. Today, technical woven fabrics designed for targeted use are of particular importance. They have replaced other materials, due to their exceptional properties, the economy of production, and their adaptability. For the production of technical fabrics, current research is moving in the direction of discovering and improving value-added composites and raw materials with features that cannot be obtained with conventional woven fabrics (3D structures, microbiological protection in medicine). Fabrics that have a pronounced third dimension (thickness) can be woven on classic weaving machines, where two fabrics are tied together with individual warps, or weft yarns are created at the same time and thus creating a unique complete structure. The 3D-woven fabrics consisted of two groups of yarns: the warp, the weft, and the interlacing yarns of the warp or weft, which occasionally connect the upper and lower fabrics. This weaving process enables weaving on classic looms, which are often equipped with two or three warp beams with warps with different consumptions or different fabric shrinkages. To obtain the more complex structures of 3D-woven fabrics, a major reconstruction of weaving machines is required; up to a completely new design of machine, with major changes to the weaving system [2,3]. Three-dimensional woven structures can be divided into orthogonal, angle interlock, multilayer, through-thickness, and layer-to-layer structures, and they can be fabricated with a conventional or modified weaving loom, as well as with a 3D weaving loom [4,5,6]. Fabrics are being researched increasingly intensively, as one of the components of flexible multi-layer materials for various purposes, such as medicine, construction, transportation, household, sports, etc. [7,8]. Technical fabrics that are intended for medical use in hospitals and other health institutions differ from each other in many parameters. Today’s production of medical fabrics is directed towards the production of different structures with high added value, such as protective and smart fabrics [9]. This group of fabrics includes sanitary material, fabrics for therapeutic and implantation purposes, as part of medical aids, special clothing, etc. Medical fabrics are most often used in contact with the body, so they must be non-toxic, non-allergenic, and non-carcinogenic. The application of fabrics in medicine is extremely broad, and according to their purpose, they can be divided into three basic groups: woven fabrics for external use for therapeutic purposes (gauzes, bandages of various shapes, compresses, and tampons); fabrics as implant substitutes (artificial blood vessels, heart valve prostheses, eye lenses); and other textile materials (protective clothing and footwear, fabrics for packing equipment in hospital sterilization, bedding, curtains, wiping accessories) [10,11,12,13].

Textiles used for packaging in hospital sterilization must meet the following conditions: an effective microbial barrier; the possibility of chemical and thermal sterilization; non-toxicity; good physical and mechanical properties; and dimensional stability [14,15]. Reusable textiles as packaging materials in sterilization are environmentally friendly, as washing and sterilization enable their reuse. The time period of use depends primarily on the effectiveness of the microbial barrier and mechanical damage [16,17]. The specific purpose of this textile is protection against microorganisms coming from healthcare workers and patients. To a large extent, they are used in operating rooms as surgical coverings. The basic task of textiles is to separate the anesthesia area from the operating room, in order to prevent the transfer of bacteria from the skin to the wound [18,19,20,21]. The microbial barrier is a basic cover or container that protects the medical material from re-contamination after sterilization. The microbial barrier system should provide protection against the penetration of microorganisms and maintain product sterility until the moment of use. An effective microbial barrier is required in packaging materials in sterilization, operating gowns, hospital sheets, compresses, etc. Packaging textiles in sterilization are used for the internal packaging of medical instruments or for external packaging as dust protection. Textiles undergo a washing process before use [22,23]. The specification of packaging materials and handling conditions are defined by EN ISO 11607 “Packaging for terminally sterilized medical devices”. “Packaging” consists of a microbial barrier system (SMB) and protective packaging. Packaging protects the sterilized material from contamination after sterilization, until use in the operating room. When packing items, it is necessary to pay attention to the mass, external shape, sharp edges, or protruding parts of instruments and accessories. The procedure for the sterilization unit is as follows: selection of packaging; packaging; sterilization of the package; storage of the package; shipment of the package to the operating room; and finally, in sterile conditions, taking the contents out of the package, without contamination [24,25].

Thermal decomposition starts at 400 °C. It is self-extinguishing and harmless to human and animal health. Exposure to aramid does not cause tumors or irritation of the skin or the respiratory system, according to research conducted on rats and humans [26].

The woven fabrics used today for packing surgical materials in the medical sterilization unit are often Tencel, cotton, or cotton/polyester, and woven in plain, twill, or atlas weave. Laminates are less often used, because of their cost. Previous studies of the fabrics used as packaging material in sterilization showed that they are permeable to microorganisms. The condition of complete impermeability of a microbial barrier entails a three-layer medical laminate. A three-layer medical laminate meets all the requirements of the EN ISO 11607 standard. However, its use in hospitals is limited, due to the cost. Research into polyurethane coated fabrics has shown that a polyurethane coating enables impermeability to microorganisms. The results showed that with polyurethane coated fabrics, mechanical damage to the polyurethane surface layer occurs after the tenth washing and sterilization process. After physical damage to the surface of a polyurethane layer, the effectiveness of the microbial barrier is questionable, and its use for packaging in sterilization ends [16,27,28,29,30,31].

In order to achieve impermeability to bacteria, and at the same time the permeability of water vapor, as well as good physical and mechanical properties, a multi-layered fabric made of fibers extremely resistant to mechanical loads was designed.

This paper aimed to determine the possibility of using a 3D aramid woven fabric, with aramid providing strong microbiological protection for the fabric on the face, and with modacrylic on the back of the fabric giving exceptional softness, but also additional protection. It was expected that, due to the known durability and stability of aramid 3D fabric, it would be possible to use it as a packaging material for the effective sterilization of medical equipment. For this purpose, the microbial barrier permeability of the developed 3D fabric was tested. The application of the mentioned material is in operating rooms and for packing surgical material for sterilization in the medical sterilization unit. Packaging fabrics are exposed during hospital sterilization to damage from being pierced by the medical devices they protect. The puncture resistance of the fabric for this group of fabrics is often overlooked. In this work, we tried to develop fabrics made from aramid fibers and with new structures that have not previously been used for packaging medical devices.

## 2. Materials and Methods

### 2.1. Basic Characteristics of Yarns and Woven Fabrics

Six woven fabric samples, developed within the research project of the Croatian Science Foundation, project code: IP-2018-01-3170, at the University of Zagreb Faculty of Textile Technology, were used. The basic parameters of the fabric samples are shown in Table 1.

**Figure 1:**
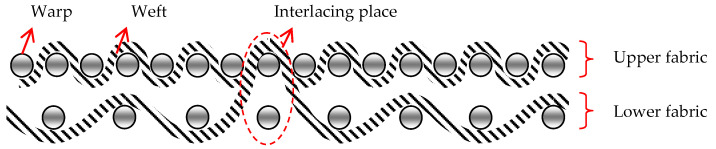
Cross-section of 3D fabric marked “pp”.

**Figure 2:**
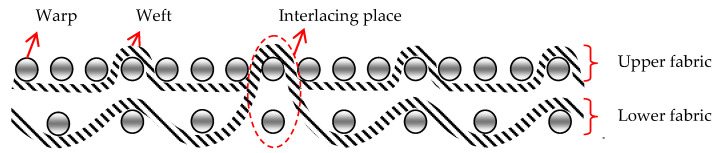
Cross-section of 3D fabric marked “tp”.

Cross-section of 3D fabric marked “pp” and “tp” shows Figure 1 and Figure 2

### 2.2. Microscope Analysis

Microscopic analysis of the surface of the 3D-woven fabrics and their 3D structure was performed using a Digital Microscope Dino-Lite Edge–5 megapix AM7115MZT, with a polarizing filter and 20× magnification.

Table 2 shows microscopic images magnified 20×, from the face and back of the fabric, according to the markings. The upper fabric, which forms the face of the aramid fiber fabric with a higher density, has a uniform surface. The lower-density cotton and modacrylic lower fabric have a pleated surface, created by interlacing with the upper fabric. The surface folds of the fabric, created by the weave, create a gap between the fabrics that is filled with air. By increasing the density of the weft, the folds become denser and more frequent, with a smaller air volume, which affects the volume, thickness, and weight of the fabric.

**Figure 3:**
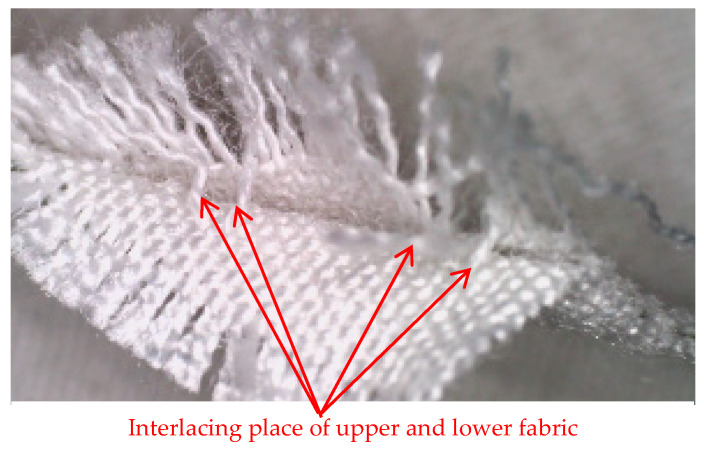
Cross-section of a 3D-woven fabric, marked “1pp” (magnification 20×).

Figure 3 shows a cross-section of a 3D-woven fabric with the plain weave, with the places where the upper and lower fabric interlace highlighted.

### 2.3. Description of the Laboratory Weaving Machine

**Figure 4:**
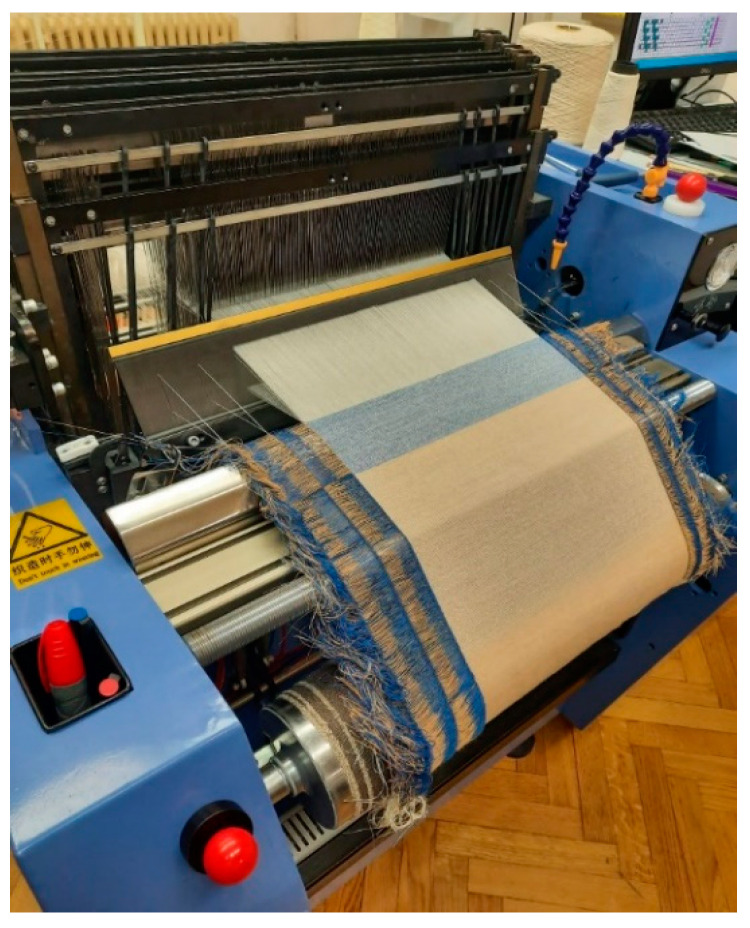
Laboratory weaving machine.

The newly developed fabric was woven on a laboratory machine (Figure 4) with the following characteristics:

- Weft entry with one rigid weave bar.
- Automatic control with a CAD/CAM weaving system.
- Weft attachment device with an adjustable application force.
- Maximum width of the base 50 cm.
- Number of wefts per minute 25–40.
- Maximum number of sheets 20.
- Automatic, electronic weft selector.
- Number of jobs for weft windings: 8.
- Base release device.
- Automatic regulation of warp tension.
- Fabric-pulling device with adjustable weft density.
- Computer and specialized sample design software.
- Manufacturer and Model: Fanyuan Instrument DW598.

### 2.4. Microbial Barrier Properties

Medical instruments, accessories, and bandage materials must be sterilized in a packaged manner, as unpackaged or unpacked materials cannot be transported or stored after sterilization. The most important function of the packaging material is to protect the packaged sterile material against contamination [17,22,23].

**Figure 5:**
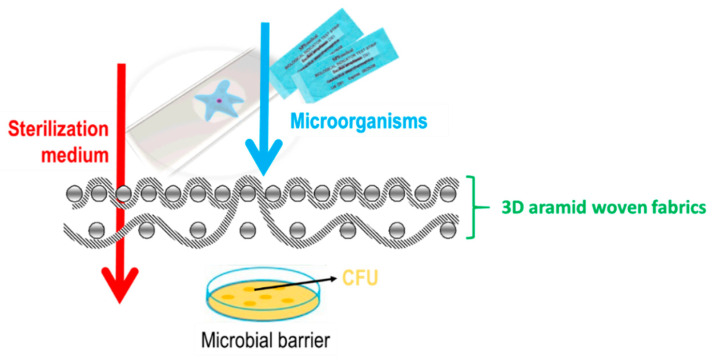
Microbial barrier.

The microbial barrier must protect the user from the penetration of microorganisms and maintain the sterility of the product until the moment of use, i.e., it must be permeable to the sterilization medium and impermeable to microorganisms, Figure 5. The purpose of this research was to test the effectiveness of the microbial barrier system of the developed 3D-woven fabrics.

**Figure 6:**
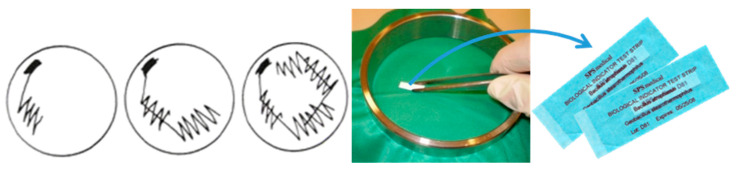
Illustration of the application of bacterial spores of *Geobacillus Stearothermophilus* and *Bacillus Atrophaeus* to the sample test field.

With cooperation between the Faculty of Textile Technology and Faculty of Medicine of the University of Zagreb, and the Clinical Hospital Center Zagreb-Rebro, in real hospital conditions, a new method of testing the microbial barrier of medical textiles had previously been developed: A washed sample measuring 22 × 22 cm is fixed in a device for testing the effectiveness of the microbial barrier and then packed into sterilization bags. It is sterilized in a steam sterilizer at 134 °C for 5 min. After sterilization, the bag with the tested sample is opened and bacterial spores are carefully applied to the sample on the upper side (face), without the possibility of contaminating the lower side (back). Bacterial spores of the genus Bacillus (*Geobacillus Stearothermophilus* and *Bacillus Atrophaeus*) were the only microorganisms used in dry form. A special feature of this work was precisely that the microorganisms were used in dry form, while suspensions of different types of microorganisms have been used for similar tests. Bacterial spores of *Geobacillus Stearothermophilus* and *Bacillus Atrophaeus* were applied to the test field of the textile sample with sterile tweezers, with an exact order of movements: left–right, up–down, and at an angle of 45° (Figure 6). After that, the biological indicator stick was turned and the procedure was repeated in the same order. In this way, extreme contamination of the textile sample was imitated [17,28,29,30].

This was followed by incubation for 24 h. After incubation, the sample was turned upside down. An imprint was taken with a CT3P agar impression plate; first of the back, then the sample was turned, and an imprint was taken of the face. After taking imprints, the agar impression plates were placed in an incubator at 35 °C for 72 h. After 72 h, the number of bacterial colonies obtained from the face and back was read [17,28,29,30].

### 2.5. Bursting Strength of the Woven Fabric Using a Steel Ball

The deformation of materials, caused by bursting, is a common phenomenon in the case of stress at the corners and in protrusions of fabrics, created by folding or pressing various objects on the woven fabric. The use of woven fabrics in the transport, storage, or protection of medical devices leads to their stress and possible damage. Most often, these are multidirectional stresses at the point of contact between the fabric and metal medical devices with sharp or blunt surfaces. To evaluate a fabric’s resistance to such damage, it is necessary to achieve test conditions that are most similar to the real ones, which usually consist of spatial (multidirectional) deformation.

**Figure 7:**
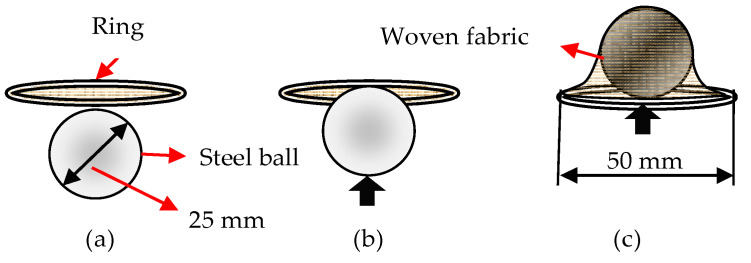
The process of bursting the fabric by punching with a steel ball: (**a**) placing fabric in the ring, (**b**) beginning the strain, and (**c**) strain immediately before the ball burst through the fabric.

Fabric testing was performed by bursting with a steel ball on a tensile tester from Apparecchi Branca S.A., according to HRN EN 12332-1:2003, see Figure 7.

### 2.6. Washing and Sterilization

The process of washing the 3D-woven fabric samples was carried out in a continuous washing machine (JENSEN brand, Gent, Belgium). The washing conditions are shown in Table 3. After washing, the samples were sterilized in a Selectomat PL MMM steam sterilizer (Münchener Medizin Mechanik, Planegg, Germany). The sterilization conditions were as follows: temperature 134 °C, pressure 2.5 bar, and time 5 min.

## 3. Results and Discussion

### 3.1. Dimensional Stability

**Figure 8:**
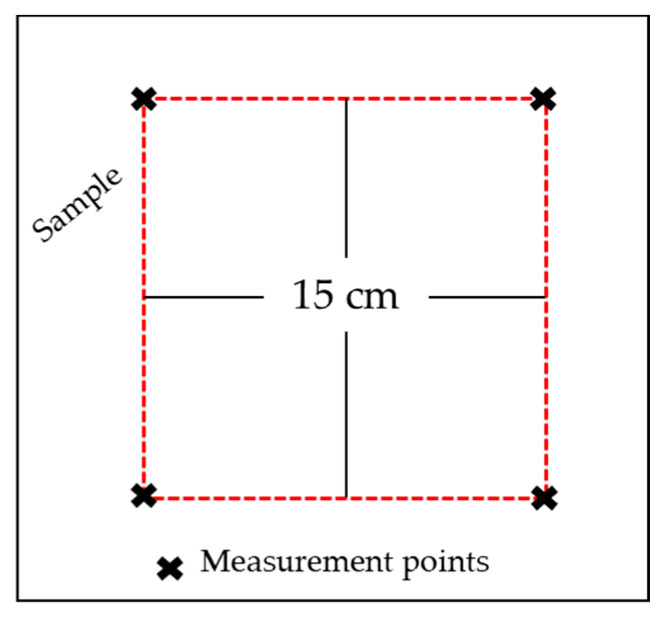
Sample labelling.

Dimensional stability was determined according to the standard test procedures HRN EN ISO 6330:2003 and HRN EN 25077:2003. To determine the change in dimensions after washing procedures, the length and width were marked on the samples, i.e., the initial dimension was 15 × 15 cm = 225 cm^2^ (Figure 8). After the washing procedures, the samples were measured again, and the percentage change in the dimensions of the samples, i.e., the surface of the samples, was calculated (Table 4).

The results after the washing process showed a change in dimensions for all 3D-woven fabric samples. The samples of 3D-woven fabric with the mark “pp” in the plain weave did not show a difference in their change in density, which amounted to −3% in all three samples. Samples marked “tp” showed a mutual difference in dimensions, i.e., lower densities had a greater shrinkage. The fabric with the highest density (tp1) had the least shrinkage (−3%), while the fabric with the lowest density (tp3) had the most shrinkage (−5%).

Table 5 shows the results of the mass per area and thickness of the 3D-woven fabrics, according to the standard [32,33].

According to the obtained results, it could be determined that the mass and thickness of the fabric was decreased by reducing the weft density in the samples: by 35/17, 32/16, and 30/14 thread/cm, see Table 1. Samples woven in plain weave, marked “pp”, had a greater mass and thickness, with a lower CV, than samples woven in twill weave, see Table 5.

### 3.2. Microbial Barrier Permeability Results

The results of the permeability of the microbial barrier are shown in Table 6. When testing the effectiveness of the microbial barrier, due to the 3D-structure created from two different fabrics (upper aramid and lower Cotton/Modacryl), tests were performed on both sides of the 3D fabric.

**Figure 9:**
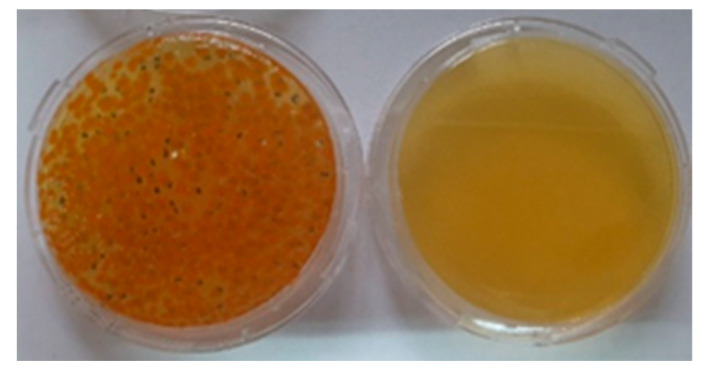
CT3P agar imprint plates of the face and back of the 3D-woven fabric, and bacterial colonies after face imprinting of the 3D-woven fabric.

The results showed that in the samples of 3D-woven fabrics 1pp, 2pp, 3pp, 1tp, and 2tp, there was no permeability of microorganisms to the inside, which means that the samples were impermeable to microorganisms (Figure 9).

One of the reasons for the impermeability of the microbial barrier was the 3D structure of the fabric. For the sample of the 3D fabric marked 3tp, there was leakage of microorganisms to the inner side of the CFU sample (144:1). Sample 3tp, in both cases of application of microorganisms, to the face (Ratio CFU 144:1) and to the reverse (Ratio CFU 166:1), showed the passage of microorganisms to the other side, Table 6. Sample 3tp of aramid woven fabric is not recommended for packaging for medical sterilization, because it does not meet the basic impermeability function of a microbial barrier. The results of the microbial barrier permeability of the 3D-woven aramid fabric after 5 and 10 washing and sterilization procedures demonstrated the impermeability of the microbial barrier for all samples. The trend of higher retained microorganisms on the back was the same as after the first washing and sterilization, see Table 6.

As the material under consideration is a 3D-woven aramid fabric, it was necessary to investigate how the microbial barrier behaves, depending on the side of the fabric to which the microorganisms are applied. The reason for this is the two different fabric structures on the face and back of the fabric. The results showed that there was a change in the number of retained microorganisms. When applying microorganisms to the back side, an increase in the number of retained microorganisms on the surface was visible. The crease on the surface of the fabric maintains a permanent gap between the fabrics and, thus, prevents the penetration of microorganisms from one side of the 3D-woven fabric to the other side. By reducing the density of fabrics, the pores become larger, making it easier for microorganisms to penetrate them. At the same time, with a higher density, the folds are more numerous, with smaller volumes, but also with more numerous indentations on a certain surface of the fabric (see Table 2), which can affect the ease of penetration of microorganisms. The fabric weave can also affect the penetration of microorganisms, especially at the interlacing of the upper and lower fabric, and where a dent is created on the surface of the fabric. The binding yarn interlaces from one fabric to another, and its tension creates larger pores in that segment of the fabric, which can allow an easier passage of microorganisms. According to the results shown in Table 3, sample “3tp” was the only fabric that did not meet the complete impermeability to microorganisms. The reason for this was it having the lowest density of the fabrics (44 threads/cm), as well as a twill weave, which creates larger pores than a plain weave.

### 3.3. Results of the Bursting of Fabrics with a Steel Ball

**Figure 10:**
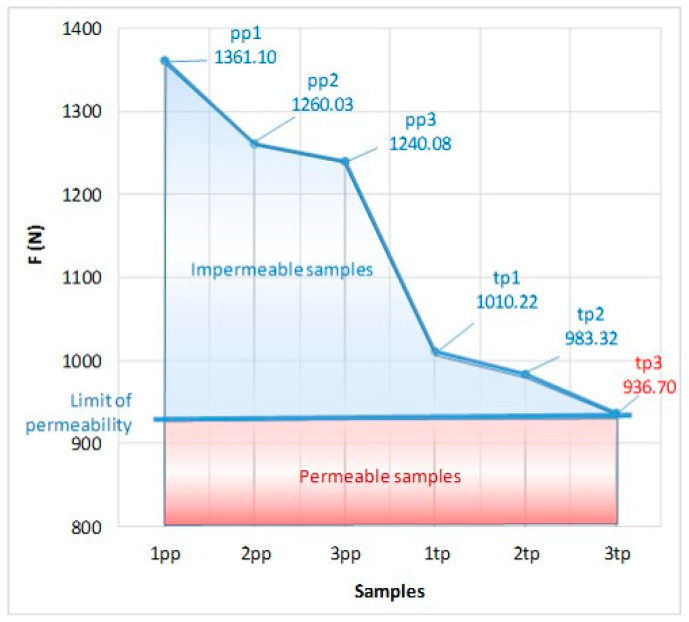
Bursting of woven fabrics according to the pattern, with surfaces of permeability/impermeability highlighted, and the limit of permeability of microorganisms through the fabric.

Utilizing the test results, the influence of the fabric density and weave on the steel ball bursting force could be determined, see Table 7. Samples marked “pp” were woven in plain weave and had higher bursting forces than the samples marked “tp#” woven in twill weave. Thus, it could be concluded that interlacing with a higher number of warps and wefts (the plain weave has the maximum number) created a greater resistance when the ball burst the fabric compared to the K3/1 twill weave, which had twice as many. Likewise, a higher fabric density provided greater bursting forces when bursting through both weaves. Figure 10 shows the surfaces of the impermeable and permeable fabric samples. The permeability limit indicated that all samples labelled “pp”, as well as samples tp1 and 2tp, were impermeable. Only sample 3tp had a low ball bursting force that was in the permeability range.

**Figure 11:**
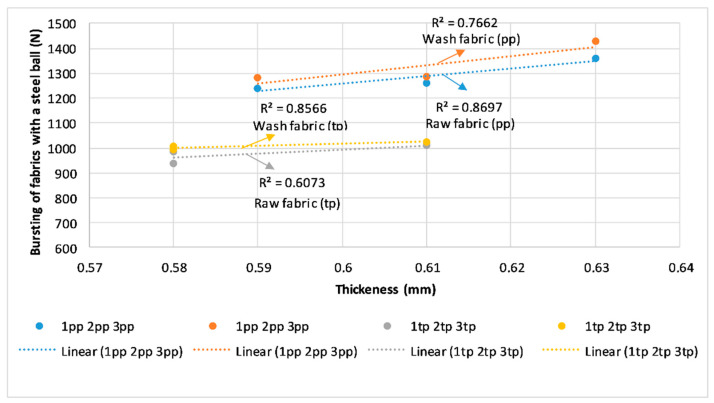
Coefficient of determination and regression between the bursting of woven fabrics (N) and thickness of the fabrics (mm).

The direction of the correlation coefficient of determination and regression between the bursting of woven fabrics and the thickness of the fabrics is shown in Figure 11. The correlation coefficient of determination was relatively large and ranged from R^2^ = 0.6073 for raw fabric in twill weave, marked “tp”, to R^2^ = 0.8697 in the case of raw fabric in plain weave, marked “pp”. Fabrics woven in plane weave had higher breaking forces, greater thickness, but also a higher coefficient of determination than those woven in twill weave. This demonstrates the advantage of weaving 3D fabrics in plain weave for the upper and lower fabric, if one wants to achieve greater resistance to punching forces and a greater thickness of the fabric.

**Figure 12:**
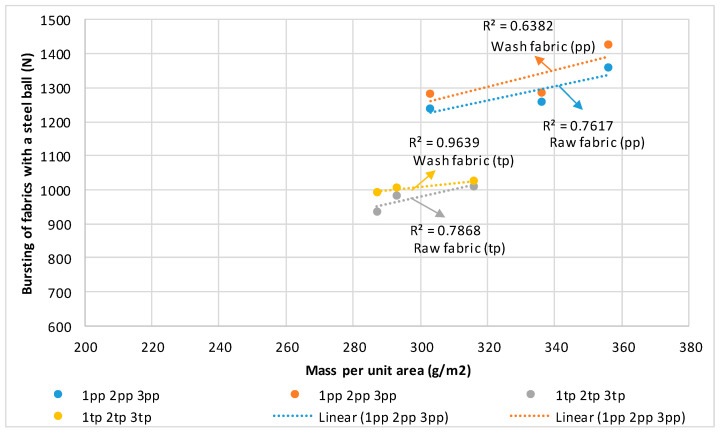
Directions of the coefficient of determination and regression between the bursting of woven fabrics (N) and mass per unit area (g/m^2^).

The directions of the correlation coefficient of determination and regression between the bursting of woven fabrics and mass per unit area are shown in Figure 12. The correlation coefficient of determination was relatively large, and ranged from R^2^ = 0.6382 for raw fabric in plain weave, marked “pp”, to R^2^ = 0.9639 in the case of raw fabric in twill weave, marked “tp”.

## 4. Conclusions

According to the results obtained, the following can be concluded:

- The new structures of 3D-woven aramid fabric provide an effective microbial barrier, and their use is justified in medicine, as packaging for sterile materials.
- The 3D-woven fabric in plain weave, in the upper and lower fabric, provided an effective microbial barrier, i.e., there was no penetration of microorganisms from the front to the back and vice versa.
- Changing the density of the 3D-woven fabric did not affect the penetration of microorganisms, which demonstrates their efficiency and economy.
- The 3D-woven fabric with a weft density of 48 and 52 weft/cm in twill weave in the upper fabric and plain weave in the lower fabric formed an effective microbial barrier.
- The 3D-woven fabric with a weft density of 44 weft/cm in twill weave in the upper fabric (face) and plain weave in the lower fabric (back) did not demonstrate complete protection from the penetration of microorganisms from the face to the back and the back to the face of the fabric.
- The developed 3D-woven aramid fabrics: 1pp, 2pp, 3pp, 1tp, and 2tp can, due to their effective microbial barrier and other protective properties, be used for packaging in medical sterilization.
- Sample 3tp is not recommended for packaging in medical sterilization because it does not have an adequate microbial barrier and does not meet the mandatory impermeability conditions for microorganisms.
- The developed 3D-woven aramid fabrics demonstrated exceptional resistance to bursting.
- Plain embroidery had a higher number of warp and weft interlacing points (fabrics marked “pp”) compared to the twill weave K3/1 (twice as many) and provided greater resistance to bursting by a steel ball.

In conclusion, the newly developed 3D fabrics made of aramid (upper fabric, face) and modacrylic (lower fabric, back) fibers in a plain weave, as well as in twill with higher densities, provide complete protection against microorganisms, exceptional stability, and resistance to multidirectional stresses, and are recommended as packaging materials for medical sterilization.

## Figures and Tables

**Table 1:** Basic parameters of the 3D-woven fabrics.

Samples	Woven Fabric	Weave Upper Fabric-Lower Fabric	Density Warp-Weft (Thread/cm)	Yarn Fineness (tex)		Yarn Composition	Mass Per Unit Area (g/m^2^)	Thickness (mm)
				Warp	Weft	Warp and Weft		
1pp ^1^	Upper	Plain	40-35	12.5 × 2	16.7 × 2	95.0% M-aramid Conex NEO 5.0% P-aramid Twaron	356	0.63
	Lower	Plain	20-17	12.5 × 2	25	45.0% Cotton Long Stapel Combed 55.0% Modacrylic Sevel FRSA/L		
2pp	Upper	Plain	40-32	12.5 × 2	16.7 × 2	95.0% M-aramid Conex NEO 5.0% P-aramid Twaron	336	0.61
	Lower	Plain	20-16	17 × 2	25	45.0% Cotton Long Stapel Combed 55.0% Modacrylic Sevel FRSA/L		
3pp	Upper	Plain	40-30	12.5 × 2	16.7 × 2	95.0% M-aramid Conex NEO 5.0% P-aramid Twaron	303	0.59
	Lower	Plain	20-14	17 × 2	25	45.0% Cotton Long Stapel Combed 55.0% Modacrylic Sevel FRSA/L		
1tp ^2^	Upper	Twill 3/1	40-35	12.5 × 2	16.7 × 2	95.0% M-aramid Conex NEO 5.0% P-aramid Twaron	316	0.61
	Lower	Plain	20-17	17 × 2	25	45.0% Cotton Long Stapel Combed 55.0% Modacrylic Sevel FRSA/L		
2tp	Upper	Twill 3/1	40-32	12.5 × 2	16.7 × 2	95.0% M-aramid Conex NEO 5.0% P-aramid Twaron	293	0.58
	Lower	Plain	20-16	17 × 2	25	45.0% Cotton Long Stapel Combed 55.0% Modacrylic Sevel FRSA/L		
3tp	Upper	Twill 3/1	40-30	12.5 × 2	16.7 × 2	95.0% M-aramid Conex NEO 5.0% P-aramid Twaron	287	0.58
	Lower	Plain	20-14	17 × 2	25	45.0% Cotton Long Stapel Combed 55.0% Modacrylic Sevel FRSA/L		

^1^ pp, plain/plain; ^2^ tp, twill 3/1/plain.

**Table 2:** Microscopic image of the surface of 3D-woven fabrics, by sample.

Woven Fabric	Upper Fabric (Face of the 3D Fabric)	Lower Fabric (Back of the 3D Fabric)	
1pp	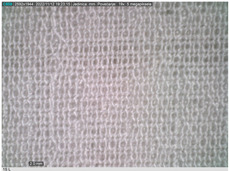	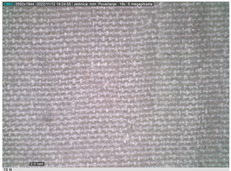	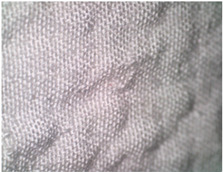
2pp	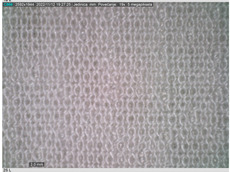	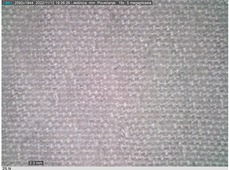	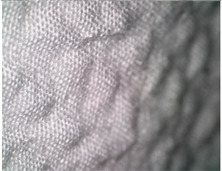
3pp	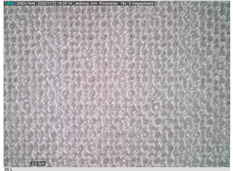	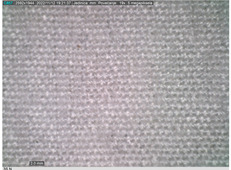	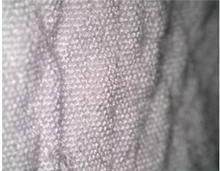
1tp	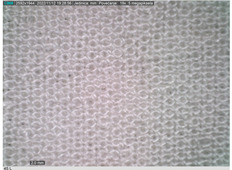	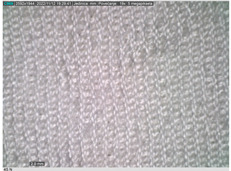	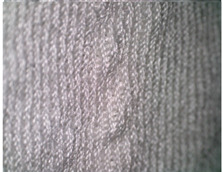
2tp	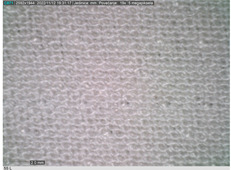	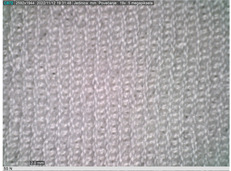	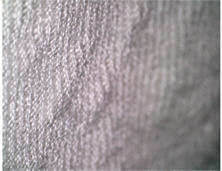
3tp	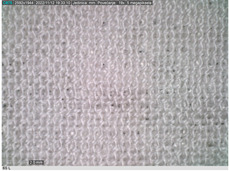	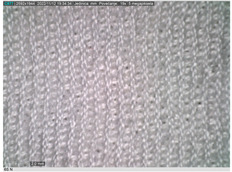	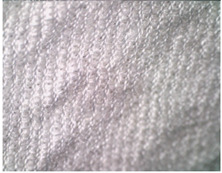

**Table 3:** Washing parameters [16,28].

Washing Solution	Disinfecting Agent	Temperature, °C	Bath Ratio
0.7 g/kg Ce 2.5 g/kg Ca	4 g/kg Cc	60	1:5

Commercial names of all products are not given, due to the secrecy of the participant laundry and the impartiality of the research. Ca—Polycarboxylate (<5%), sodium hydroxide (10–20%). Cc—Etoxylated fat alcohol <C15 and <5EO (25–30%), solvent, 2-propanol, methanol (0.1–0.25%), amphoteric surfactants (1–2%), additives (0.1–0.25%). Ce, formic acid (50–100%). Deformations of materials caused by bursting are a common phenomenon in the case of stress.

**Table 4:** Results of dimensional changes.

Samples	Raw Fabric	^1^ Dimensional Change (%)		
		Warp	Weft	Washed Fabric
1pp	225 cm^2^	15.0 cm	14.5 cm	217.5 cm^2^ −3%
2pp	225 cm^2^	15.0 cm	14.5 cm	217.5 cm^2^ −3%
3pp	225 cm^2^	15.0 cm	14.5 cm	217.5 cm^2^ −3%
1tp	225 cm^2^	14.5 cm	14.5 cm	217.5 cm^2^ −3%
2tp	225 cm^2^	14.8 cm	14.6 cm	216.1 cm^2^ −4%
3tp	225 cm^2^	14.7 cm	14.5 cm	213.2 cm^2^ −5%

^1^ Original Dimension (cm) 15 × 15 cm = 225 cm^2^.

**Table 5:** Results of mass per unit area and thickness.

Samples		Mass Per Unit Area (g/m^2^)	Thickness (mm)
1pp	Mean	356	0.63
	CV (%)	4.2	1.02
2pp	Mean	336	0.61
	CV (%)	3.8	1.46
3pp	Mean	303	0.59
	CV (%)	3.8	2.01
1tp	Mean	316	0.61
	CV (%)	4.8	1.54
2tp	Mean	299	0.58
	CV (%)	3.6	1.05
3tp	Mean	287	0.58
	CV (%)	3.9	1.85

CV, coefficient variation (%).

**Table 6:** Results of the microbial barrier permeability: average number of bacterial colonies (CFU) on the back side and the front side.

Samples	Number of Isolate	The Average Number of Bacterial Colonies on the Front Side (CFU)	The Average Number of Bacterial Colonies on the Back Side (CFU)	Ratio CFU ^1^	The Average Number of Bacterial Colonies on the Back Side (CFU)	The Average Number of Bacterial Colonies on the Front Side (CFU)	Ratio CFU
After 1 washing and sterilisation procedure							
1pp	6	154	0	-	226	0	-
2pp	6	143	0	-	196	0	-
3pp	6	133	0	-	252	0	-
1tp	6	141	0	-	182	0	-
2tp	6	126	0	-	200	0	-
3tp	6	144	1	144:1	166	1	166:1
After 5 washing and sterilisation procedures							
1pp	6	161	0	-	229	0	-
2pp	6	150	0	-	201	0	-
3pp	6	142	0	-	265	0	-
1tp	6	149	0	-	190	0	-
2tp	6	133	0	-	207	0	-
3tp	6	147	0	-	172	0	-
After 10 washing and sterilisation procedures							
1pp	6	177	0	-	233	0	-
2pp	6	152	0	-	211	0	-
3pp	6	145	0	-	272	0	-
1tp	6	150	0	-	193	0	-
2tp	6	140	0	-	215	0	-
3tp	6	151	0	-	180	0	-

^1^ CFU—Colony Forming Unit.

**Table 7:** Results of measuring breaking strength by bursting with a steel ball.

Samples		Raw Fabric		After the First Wash	
		F (N)	l (mm)	F (N)	l (mm)
1pp	Mean	1361.10	21.00	1427.11	25.66
	CV (%)	6.24	0.21	5.92	2.73
2pp	Mean	1260.03	21.33	1284.73	27.42
	CV (%)	8.05	2.71	7.55	2.01
3pp	Mean	1240.08	20.00	1318.59	26.89
	CV (%)	10.30	0.46	6.17	1.58
1tp	Mean	1010.22	20.67	1025.37	23.19
	CV (%)	2.10	2.79	4.63	1.94
2tp	Mean	983.32	20.33	1005.21	23.75
	CV (%)	7.77	2.79	7.94	1.57
3tp	Mean	936.70	20.07	992.74	24.69
	CV (%)	12.51	2.79	8.48	2.06

CV—coefficient variation (%), F—breaking strength by bursting of a steel ball through the fabric (N), l—length of movement of the ball until the fabric breaks.

## Data Availability

Not applicable.
